# Deep learning for deep learning performance: How much data is needed for segmentation in biomedical imaging?

**DOI:** 10.1371/journal.pone.0339064

**Published:** 2025-12-31

**Authors:** Junhyeok Lee, Hyungjin Chung, Minseok Suh, Jeong-Hoon Lee, Kyu Sung Choi

**Affiliations:** 1 Interdisciplinary Program in Cancer Biology, Seoul National University College of Medicine, Seoul, Republic of Korea; 2 Kim Jaechul Graduate School of AI, Korea Advanced Institute of Science and Technology, Daejeon, Republic of Korea; 3 Department of Nuclear Medicine, Seoul National University Hospital, Seoul National University College of Medicine, Seoul, Republic of Korea; 4 Department of Internal Medicine and Liver Research Institute, Seoul National University College of Medicine, Seoul, Republic of Korea; 5 Department of Radiology, Seoul National University Hospital, Seoul National University College of Medicine, Seoul, Republic of Korea; 6 Healthcare AI Research Institute, Seoul National University Hospital, Seoul, Republic of Korea; University of Marburg: Philipps-Universitat Marburg, GERMANY

## Abstract

Deep learning (DL) models are widely adopted in biomedical imaging, where image segmentation is increasingly recognized as a quantitative tool for extracting clinically meaningful information. However, model performance critically depends on dataset size and training configuration, including model capacity. Traditional sample size estimation methods are inadequate for DL due to its reliance on high-dimensional data and its nonlinear learning behavior. To address this gap, we propose a DL-specific framework to estimate the minimal dataset size required for stable segmentation performance. We validate this framework across two distinct clinical tasks: colorectal polyp segmentation from 2D endoscopic images (Kvasir-SEG) and glioma segmentation from 3D brain MRIs (BraTS 2020). We trained residual U-Nets—a simple, yet foundational architecture—across 200 configurations for Kvasir-SEG and 40 configurations for BraTS 2020, varying data subsets (2%–100% for the 2D task and 5%–100% for the 3D task). In both tasks, performance metrics such as the Dice Similarity Coefficient (DSC) consistently improved with increasing data and depth, but gains invariably plateaued beyond approximately 80% data usage. The best configuration for polyp segmentation (6 layers, 100% data) achieved a DSC of 0.86, while the best for brain tumor segmentation reached a DSC of 0.79. Critically, we introduce a surrogate modeling pipeline using Long Short-Term Memory (LSTM) networks to predict these performance curves. A simple uni-directional LSTM model accurately forecasted the final DSC, accurately forecasting the final DSC with low mean absolute error across both tasks. These findings demonstrate that segmentation performance can be reliably estimated with lightweight models, suggesting that collecting a moderate amount of high-quality data is often sufficient for developing clinically viable DL models. Our framework provides a practical, empirical method for optimizing resource allocation in medical AI development.

## Introduction

In the field of biomedical imaging, deep learning (DL) models are increasingly being adopted to extract quantitative information and support clinical decision-making [1,2]. Recently, segmentation has evolved into a powerful tool for deriving novel imaging biomarkers from routine scans by precisely delineating anatomical and pathological structures [3]. Recognizing this transformative potential, regulatory agencies have introduced streamlined approval processes for AI-enabled medical technologies [4,5].

**Fig 1:**
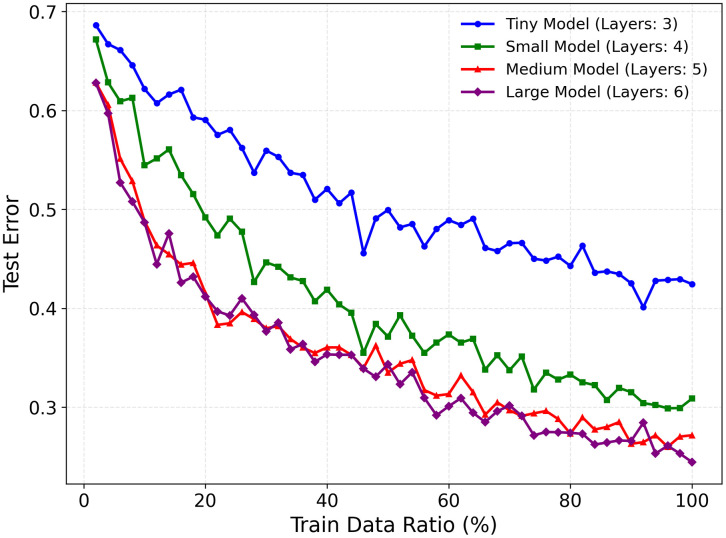
The impact of increasing dataset size and model complexity on generalization error. The graph depicts the relationship between the training dataset ratio and the corresponding test error for models of varying complexities, categorized by the number of layers.

Despite these advances, a persistent challenge in developing DL models lies in acquiring sufficient training data [6,7] (Fig 1). While larger datasets are generally presumed to enhance model generalization, real-world limitations—such as high annotation costs, patient privacy constraints, and clinical heterogeneity—often hinder large-scale data collection efforts. Biomedical datasets also frequently exhibit class imbalances [8] and institutional or demographic bias [9], further complicating the development of robust and generalizable models.

The relationship between data volume, model complexity, and performance is not linear. While increasing data or model size can improve performance, recent studies reveal that beyond certain thresholds, this scaling can induce performance degradation or plateau effects [10–12], underscoring the need for principled, task-specific planning. This raises a critical and practical question: “how much is enough?” While the pursuit of state-of-the-art performance is paramount, understanding how to achieve it efficiently is vital, particularly as not all research and clinical institutions have access to unlimited computational resources. The ability to forecast performance and estimate the point of diminishing returns can guide more sustainable and targeted research strategies.

This question is not new. As early as 2015, Cho et al. proposed an empirical framework for estimating dataset requirements in medical imaging [13], and similar questions have continued to appear in the literature [2,14,15], reflecting an enduring demand for data-efficient DL strategies. Traditional statistical power analysis methods, often used to determine minimal sample size, are ill-suited for DL-based medical imaging tasks. The complexity of high-dimensional data, annotation noise, and nonlinear model behavior deviates significantly from classical assumptions, necessitating empirical, DL-specific frameworks for data planning. While foundation models have shown promise for zero- and one-shot segmentation, they often struggle to generalize across diverse medical modalities and clinical settings without significant fine-tuning [16]. Therefore, for many specialized tasks, supervised learning remains the most practical paradigm.

To address this need, we propose a DL-specific framework to estimate the minimal dataset size required for stable segmentation performance. This approach can provide “DL-specific” guidance for study design, serving as a practical alternative to traditional statistical methods. Using two distinct proof-of-concept tasks—colorectal lesion segmentation and brain tumor segmentation—we systematically vary dataset size and model depth in residual U-Nets under controlled conditions, isolating the effects of these two core variables on performance. Our findings reinforce the perspective that acquiring moderate amounts of high-quality data may yield greater value than pursuing indiscriminate scale [17,18], a view that aligns with emerging trends in efficient model development.

## Materials and methods

### Generalizable function determination method

The universal approximation theorem asserts that neural networks with one or more hidden layers can approximate any function to arbitrary accuracy within a training dataset [19]. However, this theorem guarantees approximation capabilities only within the training environment, without ensuring generalization to unseen data. Generalization must therefore be validated using independent test datasets to evaluate performance beyond training data. Significantly, many studies emphasize that the success of neural networks stems not solely from their approximation capabilities but also from their capacity to extract meaningful features [20]. This feature-extraction ability suggests that a neural network trained on a particular task has potential predictive power on independent test datasets.

The final model performance, influenced by multiple training-related factors, arises from interactions among variables such as network architecture, initial weights, data distribution, optimization techniques, and hyperparameter configurations. These variables considerably affect both model learning capacity and generalization performance, yet accurately quantifying their individual and combined effects remains an open research challenge. To address this issue, we propose a novel framework based on “learning dynamics.” Traditional approaches from statistical learning theory, such as VC dimension [21] and Rademacher complexity [22], offer theoretical bounds on model performance but typically lack direct applicability in real-world contexts [23]. Therefore, building upon these theoretical foundations, we adopt a computational methodology supplemented by analytical insights where necessary.

### Hypothesis validation pipeline

The interplay between dataset size and model complexity significantly influences model performance. Building upon this relationship, once the function to be learned is established as generalizable, it becomes feasible to estimate the dataset size necessary to achieve clinically relevant performance. This estimation employs a predictive algorithm trained to capture the relationship between dataset size, model complexity, and test performance. Specifically, desired test performance is estimated by training a secondary neural network with performance metrics obtained from various combinations of dataset sizes and model complexities.

In this study, we introduce a hypothesis validation pipeline to predict performance for task-specific deep learning (DL) models. The pipeline comprises two primary components: a task model and a performance prediction model. The task model is trained on datasets of varying sizes and models with differing parameter counts, allowing exploration of how these factors impact performance for a specific task. The performance prediction model receives dataset size and model parameter count as inputs, predicting task model performance under specified conditions. This secondary model is trained to capture the “learning dynamics” of the task model, enabling predictions of generalization error based on training configurations. Integrating these components, our pipeline estimates the optimal dataset size required to meet target test error thresholds for clinical applications. This approach simplifies resource allocation for training and provides insights into how training conditions influence model generalization. Consequently, the pipeline offers a systematic methodology to optimize neural network development in biomedical imaging contexts.

### Biomedical segmentation tasks

As a proof-of-concept, we applied our framework to two distinct segmentation tasks.

#### Task 1: Colorectal cancer lesion segmentation.

We first developed a DL model for the automatic segmentation of polyps from 2D colonoscopy images. This task is critical for the early detection of colorectal cancer, a leading cause of cancer-related mortality worldwide. For this task, we utilized the Kvasir-SEG dataset [24], a publicly available collection of 1,000 polyp images and their corresponding ground truth masks. Image resolutions vary from 332 × 487–1920 × 1072 pixels. The dataset was partitioned into training (n = 700), validation (n = 100), and test subsets (n = 200). All images were standardized to a 256 × 256 pixel resolution for consistency, and ground-truth annotations were provided as binary regions of interest (ROIs).

#### Task 2: Brain tumor segmentation.

To validate the generalizability of our framework, we selected a second, more complex task: multi-class glioma segmentation from 3D brain MRI scans. We used the publicly available BraTS 2020 dataset [25], which contains multimodal 3D MRIs (T1, T1-Gd, T2, T2-FLAIR) and ground-truth masks for enhancing tumor (ET), tumor core (TC), and whole tumor (WT). For this study, we focused on the whole tumor segmentation task. The dataset was partitioned into training (n = 258), validation (n = 37), and test (n = 74) sets. All MRI volumes were co-registered, interpolated to a uniform 1 mm³ isotropic resolution, and skull-stripped. We extracted 128 × 128 × 128 voxel patches from the volumes for training to manage the computational load of the 3D data.

### Model architecture and training

**Fig 2:**
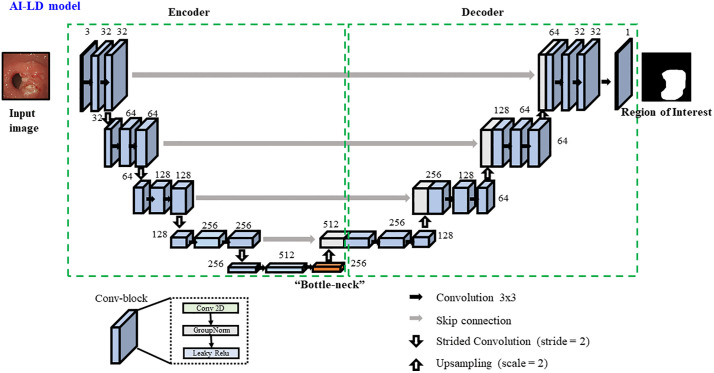
Detailed schematic of the residual U-Net architecture for lesion segmentation, exemplified by a model with a layer depth of 4. The network employs an encoder-decoder structure connected via skip connections, enhancing spatial detail retention. Encoder layers featuring residual connections in the down-sampling pathway. The image and lesion mask were obtained from the publicly available Kvasir-SEG dataset (https://datasets.simula.no/dataset/kvasir-seg).

The task-specific DL model employed a residual U-Net architecture [26,27] (Fig 2) for lesion segmentation.

#### Justification for model choice.

Our selection of the residual U-Net was a deliberate strategic decision. The U-Net architecture is a seminal and foundational model in biomedical image segmentation. Its encoder-decoder structure with skip connections has become the de facto standard and forms the backbone of countless state-of-the-art models currently in use. By focusing our investigation on a fundamental property of this core architecture—network depth—we aim to provide insights that are broadly applicable to the entire community that builds upon this framework. This focused approach allows us to isolate the impact of dataset size and model depth without introducing the confounding variables that would arise from a comparison across many disparate, complex architectures.

#### Training protocol.

The model was trained in a supervised learning framework using paired input images and manually annotated ROIs. It was trained using pixel-wise binary cross-entropy loss and optimized with the Adam optimizer [28]. Model complexity was modulated by varying the convolutional network depth (3, 4, 5, or 6 layers). For each task, we generated 50 distinct dataset sizes by sampling incremental subsets of the training data, from 2% to 100% at 2% intervals. For the brain tumor segmentation model, we used depths of 3 or 5 layers and sampled the training data from 5% to 100% at 5% intervals.

#### Performance prediction model.

**Fig 3:**
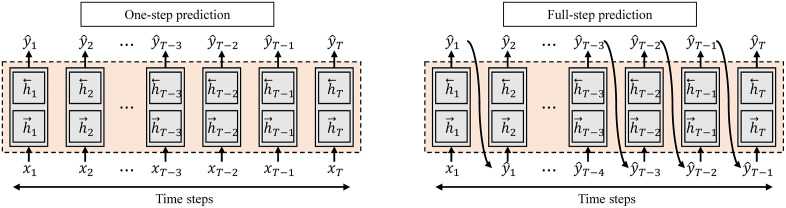
Illustrations of different prediction strategies using Long Short-Term Memory (LSTM) models: one-step prediction model, and full-step prediction model. The one-step prediction approaches independently predict outcomes at each individual time point, while the full-step prediction approach sequentially predicts multiple future outcomes, utilizing previously predicted results to inform subsequent predictions.

To forecast segmentation performance (as measured by DSC) across dataset sizes, four distinct performance prediction models based on long short-term memory (LSTM) [29] networks were developed (Fig 3):

Uni-directional one-step prediction model: This model predicts the DSC value for the next data point (t) using only the sequence of actual, observed DSC values from previous data points (1 to t-1). It makes a single forecast at each step.

Uni-directional full-step prediction model: This model is autoregressive. It first uses the observed DSC values to make an initial prediction, and then uses its own previously predicted DSCs as input to forecast all subsequent values in the sequence.

Bi-directional one-step prediction model: This model uses a bi-directional LSTM to process the input sequence of observed DSCs from both forward (past to present) and backward (future to present) directions, providing a richer context to predict the DSC value at each individual step.

Bi-directional full-step prediction model: Similar to its uni-directional counterpart, this model is autoregressive but leverages a bi-directional LSTM. It uses context from both directions to predict the entire sequence of DSC values sequentially.

All LSTM models were optimized with the Adam optimizer, using the DSC data generated by the task models as input.

### Evaluation metrics

The performance of the task-specific DL models was assessed using standard segmentation metrics, including Intersection over Union (IoU), Dice Similarity Coefficient (DSC), Recall, and Precision. DSC values from the task-specific model on the test set served as both inputs and target outputs for the performance prediction models. Predictive performance of the LSTM models was evaluated using Mean Absolute Error (MAE).

## Results

### Task 1: Colorectal lesion segmentation evaluation

**Fig 4:**
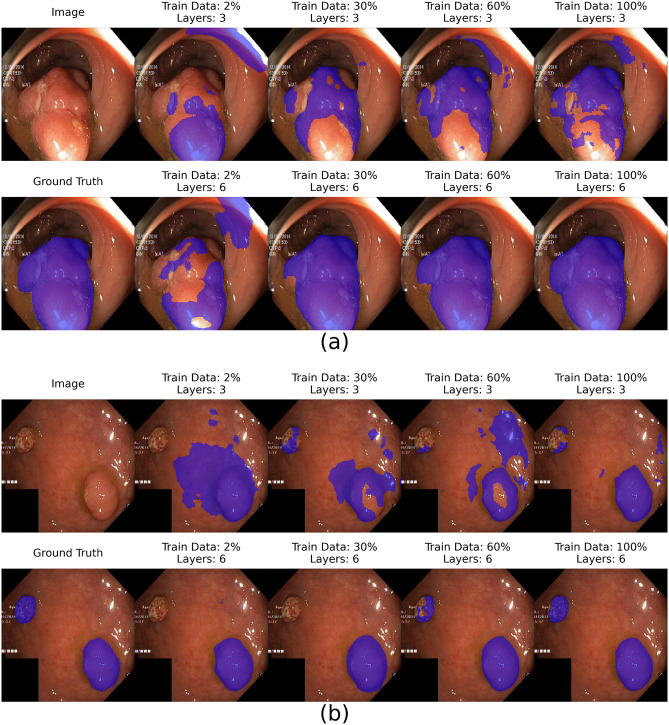
Qualitative Segmentation Results for Colorectal Polyps. Representative examples of polyp segmentation on colonoscopy images. Sub-figures (a) and (b) compare the performance of a shallow model (3 layers, top row) and a deep model (6 layers, bottom row) trained on increasing amounts of data (2%, 30%, 60%, and 100%). The predicted segmentation masks (blue overlay) become progressively more accurate and aligned with the ground truth as both training data volume and model depth increase.

**Fig 5:**
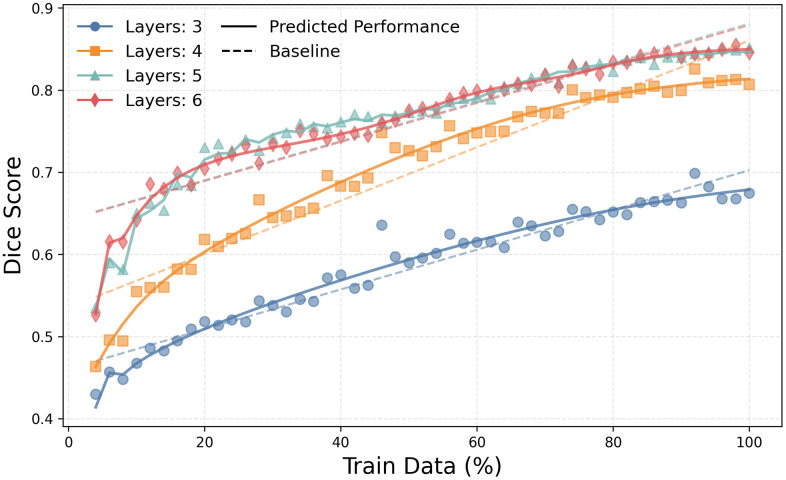
DSC Performance Prediction for Polyp Segmentation using the Uni-directional Full-step Model. Performance prediction results for the Kvasir-SEG dataset using the uni-directional full-step LSTM model. The scatter points represent the actual Dice Similarity Coefficient (DSC) scores achieved by the U-Net model for each layer depth. The solid lines show the LSTM model’s predicted performance curve, which closely tracks the actual results. The dashed lines represent a simple linear regression baseline for comparison.

S1–S4 Figs display heatmaps of segmentation metrics (IoU, DSC, Recall, Precision) on the Kvasir-SEG test set as functions of training data ratio and model depth. All metrics demonstrate improvement with larger training datasets, with deeper architectures showing pronounced gains once the training data ratio surpasses approximately 30%. The best configuration (6 layers, 100% data) achieved an IoU of 0.76, DSC of 0.86, Recall of 0.84, and Precision of 0.88. S5–S12 Figs further illustrate these trends. Fig 4 shows representative segmentation masks, where accuracy clearly improves with both increasing data and model depth. Deeper models consistently produce more refined delineations, reflecting their enhanced capacity to capture subtle details. A minor but notable observation in the performance curves (Fig 5) is the presence of small fluctuations, particularly at lower data ratios. These fluctuations are likely attributable to the stochastic elements inherent in DL training, such as random weight initialization and data shuffling, as well as the specific composition of the smaller, randomly sampled data subsets.

### Task 2: Brain tumor segmentation evaluation

**Fig 6:**
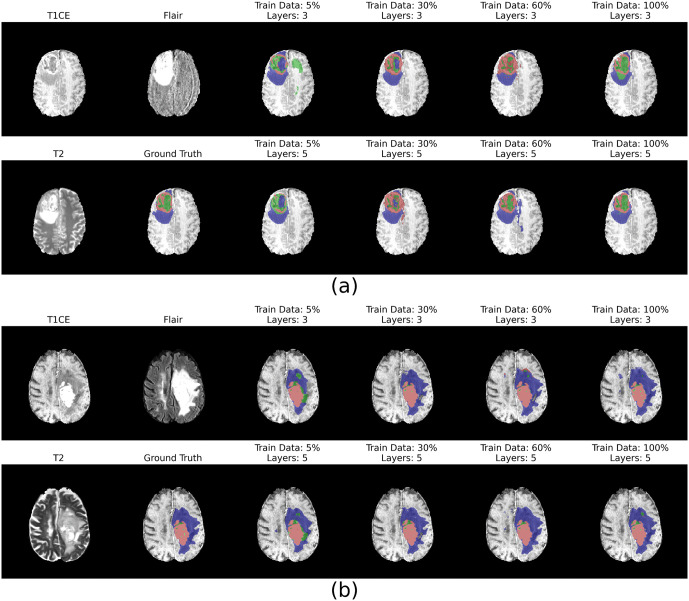
Qualitative Segmentation Results for Brain Tumors. Representative examples of whole tumor segmentation on 3D brain MRI scans. The figure illustrates how predicted tumor masks (colored overlay) improve with more data and greater model complexity. Each row compares a shallow model (3 layers) to a deep model (5 layers) trained on varying data ratios (e.g., 5%, 30%, 60%, 100%). Deeper models trained on more data produce segmentations that more accurately delineate the tumor boundaries, closely matching the ground truth.

**Fig 7:**
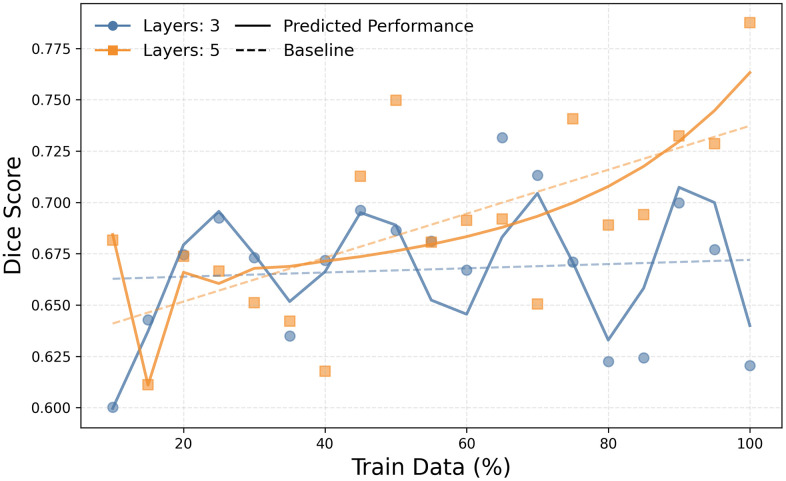
DSC Performance Prediction for Brain Tumor Segmentation using the Uni-directional Full-step Model. Performance prediction results for the BraTS 2020 dataset, generated by the uni-directional full-step LSTM model. As in Fig 6, the plot compares the actual DSC scores (scatter points) from the segmentation task with the predicted performance curve from the LSTM model (solid lines) and a linear regression baseline (dashed lines). The model accurately forecasts the performance trend, showing consistent improvement before plateauing.

To confirm the generalizability of our findings, we replicated the experiment on the BraTS 2020 dataset. Fig 6 provides qualitative examples, showing how the predicted tumor masks on MRI scans become more accurate and complete as training data and model depth increase, closely aligning with the ground truth. This visual improvement is mirrored by the quantitative results, summarized in Fig 7, which show a remarkably similar trend. Performance on the whole tumor segmentation task improved consistently with increases in both the training data ratio and model depth. The best-performing model (5 layers, 100% data) achieved a DSC of 0.79 and an IoU of 0.65. As with the Kvasir-SEG experiment, performance gains began to plateau significantly after the training data ratio exceeded 80%. The 3D nature of the task resulted in overall higher computational requirements, but the fundamental relationship between data, complexity, and performance held.

### Model complexity and training time analysis

To provide a comprehensive reference for practical applications, we analyzed the computational cost of our models. Table 1 details the parameter counts, computational complexity, and inference throughput for each model depth. As expected, all metrics increase with model depth. The number of parameters increases rapidly with model depth, leading to a substantial rise in computational demand and training duration. This analysis highlights the critical trade-off between model performance and the computational resources required to train and deploy such models.

**Table 1:** Model Complexity and Computational Cost Analysis for 2D Segmentation Task (Kvasir-SEG).

Model Depth	Parameters (M)	FLOPs (M)	Throughput (FPS)
3	6.0	12.1	66.2
4	25.6	**51.2**	**15.6**
5	**103.9**	207.7	10.0
6	416.9	833.8	10.0

This table details the computational requirements of the residual U-Net architecture at varying depths. ‘Parameters’ refers to the total number of trainable weights in millions (M). ‘FLOPs’ (Floating Point Operations per Second) quantifies the computational complexity for a single forward pass, also in millions (M). ‘Throughput’ measures the inference speed in Frames Per Second (FPS). These metrics highlight the trade-off between model capacity and the resources required for training and deployment.

### Task performance prediction evaluation

Fig 5 demonstrates the predictive accuracy of the uni-directional full-step performance prediction model in estimating DSC scores for the Kvasir-SEG task. The scatter points represent actual DSC scores, while solid lines indicate the model’s prediction. The prediction model reliably extrapolates performance, closely aligning with empirically observed scores, particularly for datasets using ≥50% of the training data. This underscores the model’s reliability in forecasting segmentation performance. This stepwise predictive approach effectively captures the inherent learning dynamics, facilitating accurate DSC score forecasting. Similarly, Fig 7 shows the prediction results for the BraTS 2020 task, where the LSTM model again accurately forecasts the performance trend, showing consistent improvement before plateauing. S13–S18 Figs illustrate similarly high predictive accuracy for the other LSTM model variants across both tasks.

### Comparison of task performance prediction models

The influence of model depth on prediction accuracy was evaluated across all LSTM methods for the Kvasir-SEG dataset. As shown in Table 2, prediction errors generally decreased with increasing model depth, indicating enhanced predictive stability in deeper models. The uni-directional full-step prediction model achieved the lowest overall mean absolute error (0.0067 ± 0.0066), slightly outperforming the uni-directional one-step model (0.0075 ± 0.0076). Bi-directional models presented marginally higher mean errors. Consequently, the uni-directional full-step prediction model demonstrated the most robust prediction capability overall. A similar analysis on the BraTS 2020 dataset (Table 3) showed the uni-directional one-step prediction model achieving the lowest MAE (0.0153 ± 0.0157).

**Table 2:** Predictive Performance on Colorectal Polyp Segmentation.

Prediction method	Model depth				Average
	3	4	5	6	
Uni-directional one-step prediction model	**0.0085 ± 0.0085**	0.0084 ± 0.0074	0.0082 ± 0.0089	0.0049 ± 0.0043	0.0075 ± 0.0076
Uni-directional full-step prediction model	**0.0085 ± 0.0085**	0.0084 ± 0.0075	**0.0050 ± 0.0041**	**0.0049 ± 0.0042**	**0.0067 ± 0.0066**
Bi-directional one-step prediction model	0.0088 ± 0.0082	**0.0084 ± 0.0073**	0.0063 ± 0.0059	0.0050 ± 0.0039	0.0071 ± 0.0067
Bi-directional full-step prediction model	0.0089 ± 0.0083	0.0085 ± 0.0075	0.0061 ± 0.0058	0.0050 ± 0.0039	0.0071 ± 0.0068

The table displays the Mean Absolute Error (MAE) for four different Long Short-Term Memory (LSTM) based prediction models on the Kvasir-SEG dataset. These models were trained to forecast the Dice Similarity Coefficient (DSC) of the segmentation task model. The MAE is calculated between the predicted and actual DSC scores, with results categorized by the segmentation model’s layer depth. Lower MAE values indicate higher predictive accuracy.

**Table 3:** Predictive Performance on Brain Tumor Segmentation.

Prediction method	Model depth		Average
	3	5	
Uni-directional one-step prediction model	**0.0094 ± 0.0100**	0.0211 ± 0.0180	**0.0153 ± 0.0157**
Uni-directional full-step prediction model	0.0135 ± 0.0137	0.0215 ± 0.0198	0.0175 ± 0.0175
Bi-directional one-step prediction model	0.0105 ± 0.0108	**0.0207 ± 0.0183**	0.0156 ± 0.0159
Bi-directional full-step prediction model	0.0133 ± 0.0137	0.0212 ± 0.0197	0.0173 ± 0.0174

This table presents the Mean Absolute Error (MAE) of the four prediction models for the brain tumor segmentation task using the BraTS 2020 dataset. Similar to Table 1, it measures the accuracy of the LSTM models in forecasting the U-Net’s DSC performance across different training data subsets and model depths.

## Discussion

This study introduced and validated a DL framework for estimating data and model size requirements, focusing on the relationships between dataset size, model complexity, and predictive performance. Our findings, now demonstrated across two distinct tasks—2D endoscopic polyp segmentation and 3D brain tumor segmentation—show that larger datasets and deeper models significantly enhance performance up to a point of diminishing returns. In both cases, metrics such as DSC and IoU showed consistent improvement as the training data ratio increased, but these gains began to plateau after approximately 80% of the dataset was used. This suggests that while more data is generally beneficial, a saturation point exists where the cost of further data acquisition may not be justified by the marginal performance improvement. Similarly, increasing model depth improved performance, but this improvement plateaued as complexity increased. This finding was consistent across both the 2D task (evaluated with 3–6 layers) and the 3D task (evaluated with 3 and 5 layers).

The strength of our framework lies in its validated applicability across different imaging modalities (endoscopy vs. MRI) and task complexities (2D binary vs. 3D multi-class segmentation). This suggests that the principle of modeling “learning dynamics” to predict a performance plateau is a generalizable strategy. Researchers working on other tasks, such as lung nodule detection in CT scans (e.g., LUNA16) [30] or cell segmentation in microscopy, could adopt this method to perform a preliminary analysis on a smaller data fraction to forecast the resources needed to reach a target performance level, thereby optimizing study design and resource allocation.

It is important to acknowledge the deliberate scope of our study. We focused exclusively on varying model depth within the U-Net architecture to isolate a key component of model complexity. This was a strategic choice to avoid the confounding variables that would arise from comparing different architectural families. While this provides a clean analysis of depth’s impact, it is not an exhaustive exploration of all possible model designs. Future work should undoubtedly extend this framework to other popular architectures (e.g., Transformers, Attention-based models) to see if similar learning dynamics hold.

**Fig 8:**
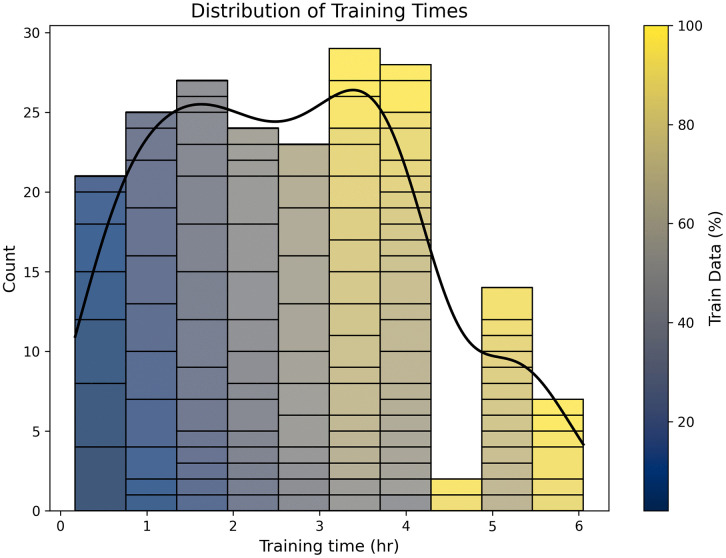
Distribution of Training Times as a Function of Training Data Ratio. The histogram illustrates the distribution of training times (in hours) for models trained with varying proportions of the dataset (train data ratio, indicated by the color gradient bar). Each bin represents counts of models completing training within specific time intervals, with darker colors indicating lower train data ratios and lighter colors indicating higher train data ratios.

Our performance prediction pipeline, using a simple LSTM, proved highly effective. It achieved a low mean absolute error (MAE < 0.007) in both tasks, accurately predicting the final DSC scores across various configurations. This provides an efficient method for optimizing model development without resorting to exhaustive trial-and-error experimentation. The training duration analysis (Fig 8) further emphasizes the need for this efficiency. While training a single model for 6 hours might seem acceptable, the cumulative time for hyperparameter tuning across hundreds of configurations becomes prohibitive. Our framework mitigates this by allowing researchers to forecast the endpoint. For resource-constrained settings, established techniques like transfer learning [31,32] and model compression [33,34] could be used in conjunction with our framework to further reduce computational demands.

Our work builds upon the understanding that traditional statistical power analyses are ill-suited for DL tasks, a point raised by others in the field [11,12]. Our empirical findings align with reports of performance saturation [12], and challenge the “more is always better” assumption. This reinforces the idea that focusing on acquiring a moderate amount of high-quality data can be more efficient than indiscriminately expanding dataset size, a philosophy that resonates with recent trends in efficient AI, such as model distillation from smaller, high-fidelity datasets [35].

A primary limitation of our study is that the proposed framework was validated on only two datasets. Although our results are promising, further validation across a more diverse range of medical imaging datasets is crucial to fully establish the framework’s generalizability and robustness. To this end, future work should prioritize evaluation on established public benchmarks; datasets such as LUNA16 [30] would serve as excellent candidates for these validation studies. Looking forward, the framework could be further enhanced by systematically analyzing other variables, such as the impact of different data augmentation strategies or optimizer choices. Furthermore, extending the prediction pipeline to leverage multimodal data—by integrating imaging with clinical reports or genomic data, for example—and to incorporate advanced transfer learning techniques represents a promising avenue for future research.

In conclusion, this study presents a systematic and empirically validated framework for optimizing dataset size and model complexity in biomedical deep learning applications. Our core contribution is a practical methodology that allows researchers to forecast segmentation performance and estimate the point of diminishing returns for data collection and model scaling. By demonstrating its effectiveness on both 2D endoscopic and 3D MRI datasets, we show that this approach offers a scalable, resource-efficient solution for model development. The simple yet powerful LSTM-based prediction model provides reliable, actionable guidance, facilitating impactful AI development by enabling a more strategic balance between performance and computational efficiency.

## Supporting information

S1 Fig The IoU values across different data ratios and model architectures. (PNG)

S2 Fig The Dice Score values across different data ratios and model architectures. (PNG)

S3 Fig The Recall values across different data ratios and model architectures. (PNG)

S4 Fig The Precision values across different data ratios and model architectures. (PNG)

S5 Fig IoU changes with increasing data ratio for different model architectures. (PNG)

S6 Fig Dice Score changes with increasing data ratio for different model architectures. (PNG)

S7 Fig Recall changes with increasing data ratio for different model architectures. (PNG)

S8 Fig Precision changes with increasing data ratio for different model architectures. (PNG)

S9 Fig IoU changes with increasing model complexity for different data ratios. (PNG)

S10 Fig Dice Score changes with increasing model complexity for different data ratios. (PNG)

S11 Fig Recall changes with increasing model complexity for different data ratios. (PNG)

S12 Fig Precision changes with increasing model complexity for different data ratios. (PNG)

S13 Fig DSC performance prediction for polyp segmentation using the uni-directional one-step model. Performance prediction results generated by the uni-directional one-step LSTM model for the Kvasir-SEG dataset. The plot compares the actual DSC scores (scatter points) from the segmentation task with the model’s predicted performance (solid lines) and a linear regression baseline (dashed lines). This one-step model predicts each data point based on the sequence of preceding actual values.
(TIF)

S14 Fig DSC performance prediction for polyp segmentation using the bi-directional one-step model. Performance prediction results from the bi-directional one-step LSTM model for the Kvasir-SEG dataset. The plot elements are consistent with previous figures: actual DSC scores (scatter points), predicted performance (solid lines), and a linear regression baseline (dashed lines). This model leverages both past and future data context to predict each point independently.
(TIF)

S15 Fig DSC performance prediction for polyp segmentation using the bi-directional full-step model. Performance prediction results from the bi-directional full-step LSTM model for the Kvasir-SEG dataset. The graph displays the actual DSC scores (scatter points) against the autoregressive predicted performance curve (solid lines) and a linear regression baseline (dashed lines). This model uses bi-directional context and its own previous predictions to forecast the entire performance trajectory.
(TIF)

S16 Fig DSC performance prediction for brain tumor segmentation using the uni-directional one-step model. Performance prediction results generated by the uni-directional one-step LSTM model for the BraTS 2020 dataset. The plot compares the actual DSC scores (scatter points) from the segmentation task with the model’s predicted performance (solid lines) and a linear regression baseline (dashed lines). This one-step model predicts each data point based on the sequence of preceding actual values.
(TIF)

S17 Fig DSC performance prediction for brain tumor segmentation using the bi-directional one-step model. Performance prediction results from the bi-directional one-step LSTM model for the BraTS 2020 dataset. The plot elements are consistent with previous figures: actual DSC scores (scatter points), predicted performance (solid lines), and a linear regression baseline (dashed lines). This model leverages both past and future data context to predict each point independently.
(TIF)

S18 Fig DSC performance prediction for brain tumor segmentation using the bi-directional full-step model. Performance prediction results from the bi-directional full-step LSTM model for the BraTS 2020 dataset. The graph displays the actual DSC scores (scatter points) against the autoregressive predicted performance curve (solid lines) and a linear regression baseline (dashed lines). This model uses bi-directional context and its own previous predictions to forecast the entire performance trajectory.
(TIF)
