# Supplementary figures and images for: Deep learning for deep learning performance: How much data is needed for segmentation in biomedical imaging?
Source: PLoS One. 2025 Dec 31;20(12):e0339064. doi: 10.1371/journal.pone.0339064 (PMC12755799; doi:10.1371/journal.pone.0339064)

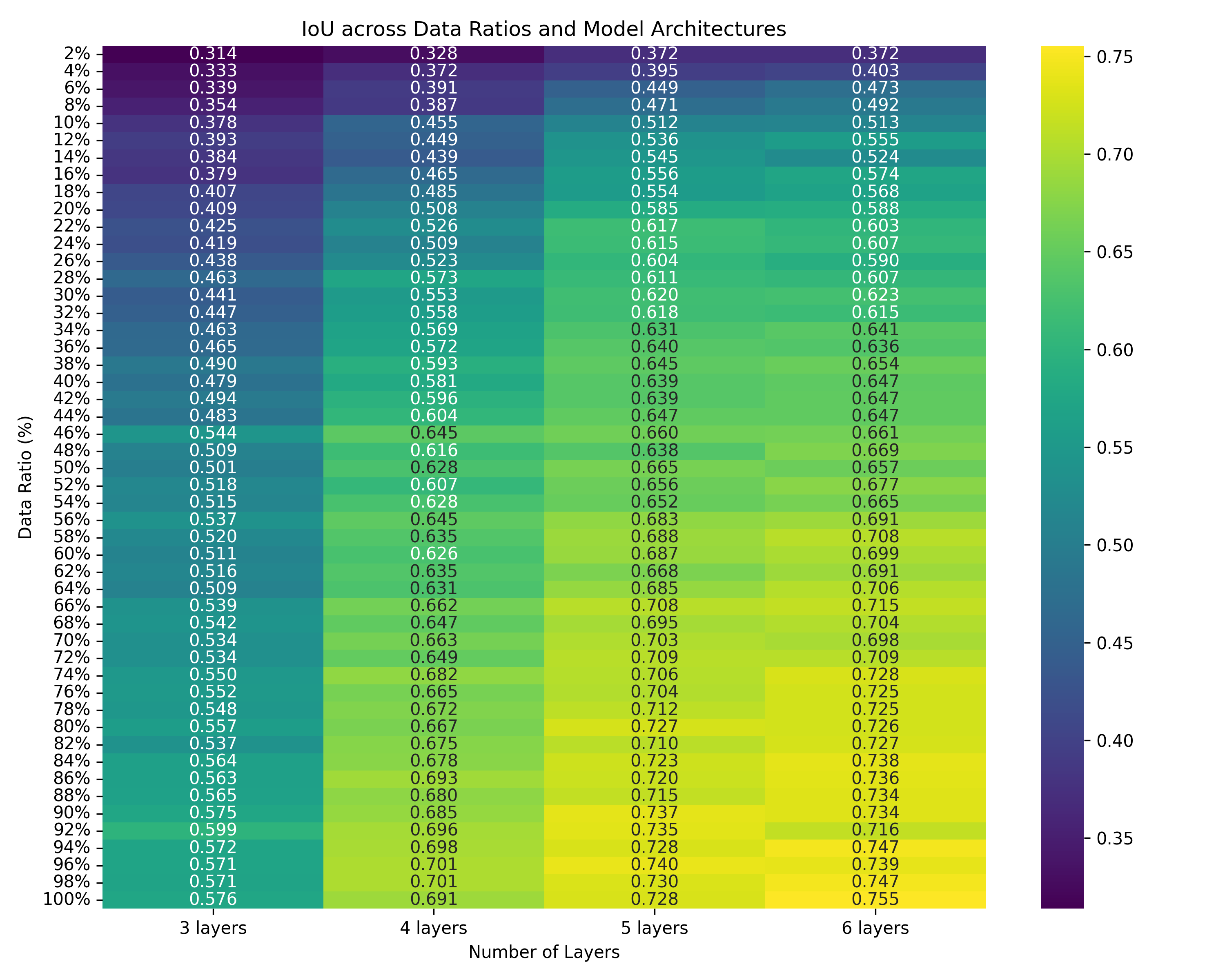

Supplement: S1 Fig — (PNG) [file pone.0339064.s001.png]

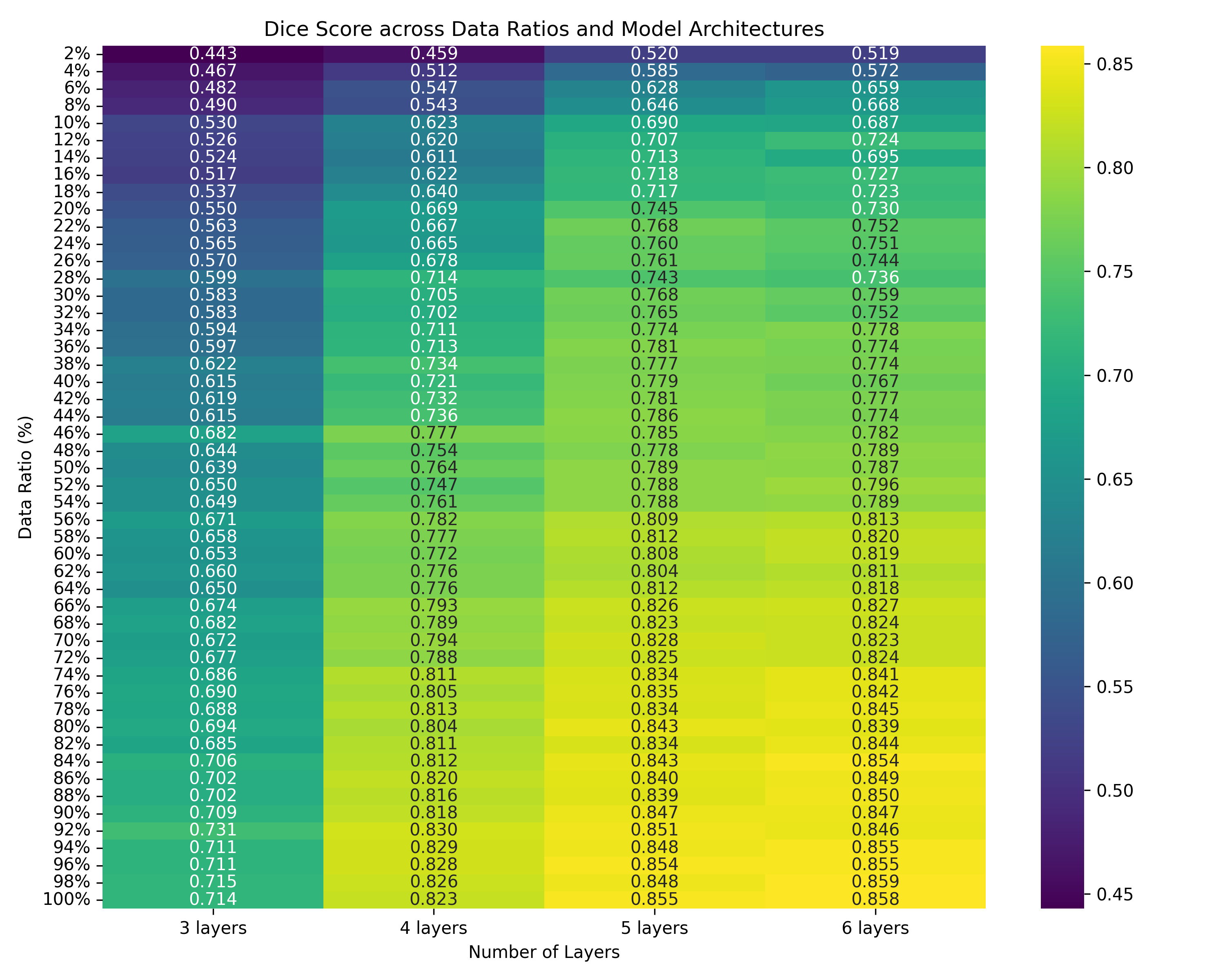

Supplement: S2 Fig — (PNG) [file pone.0339064.s002.png]

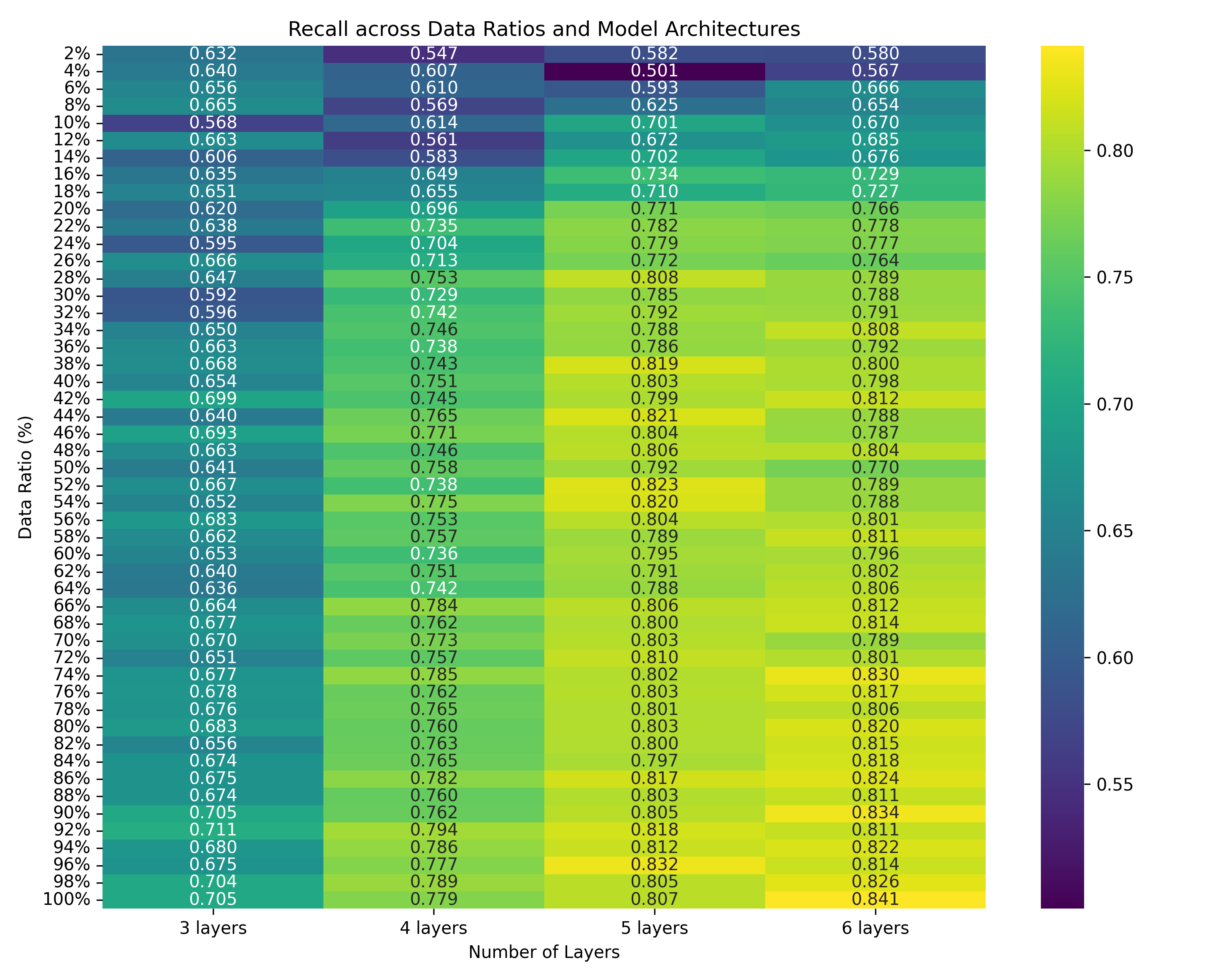

Supplement: S3 Fig — (PNG) [file pone.0339064.s003.png]

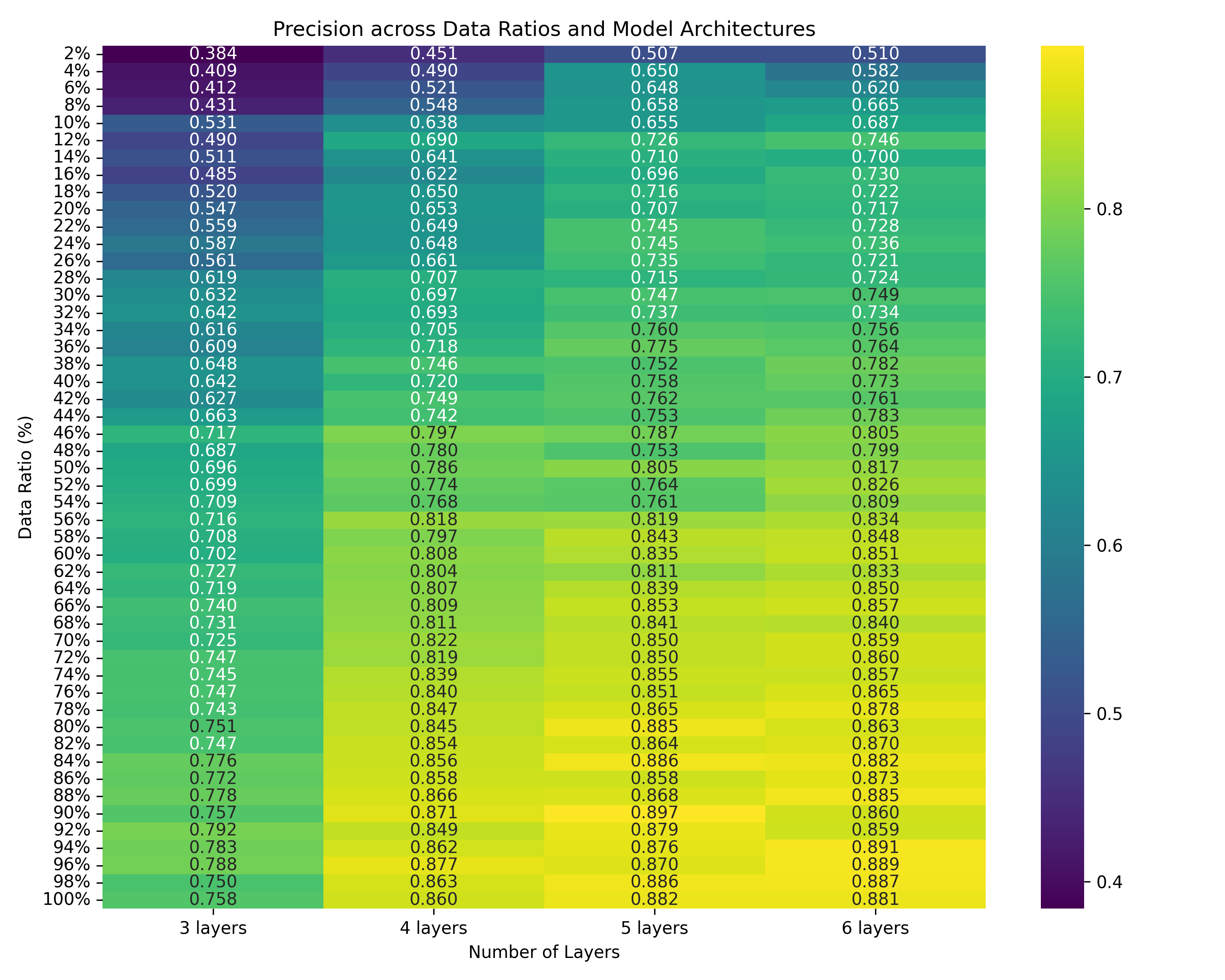

Supplement: S4 Fig — (PNG) [file pone.0339064.s004.png]

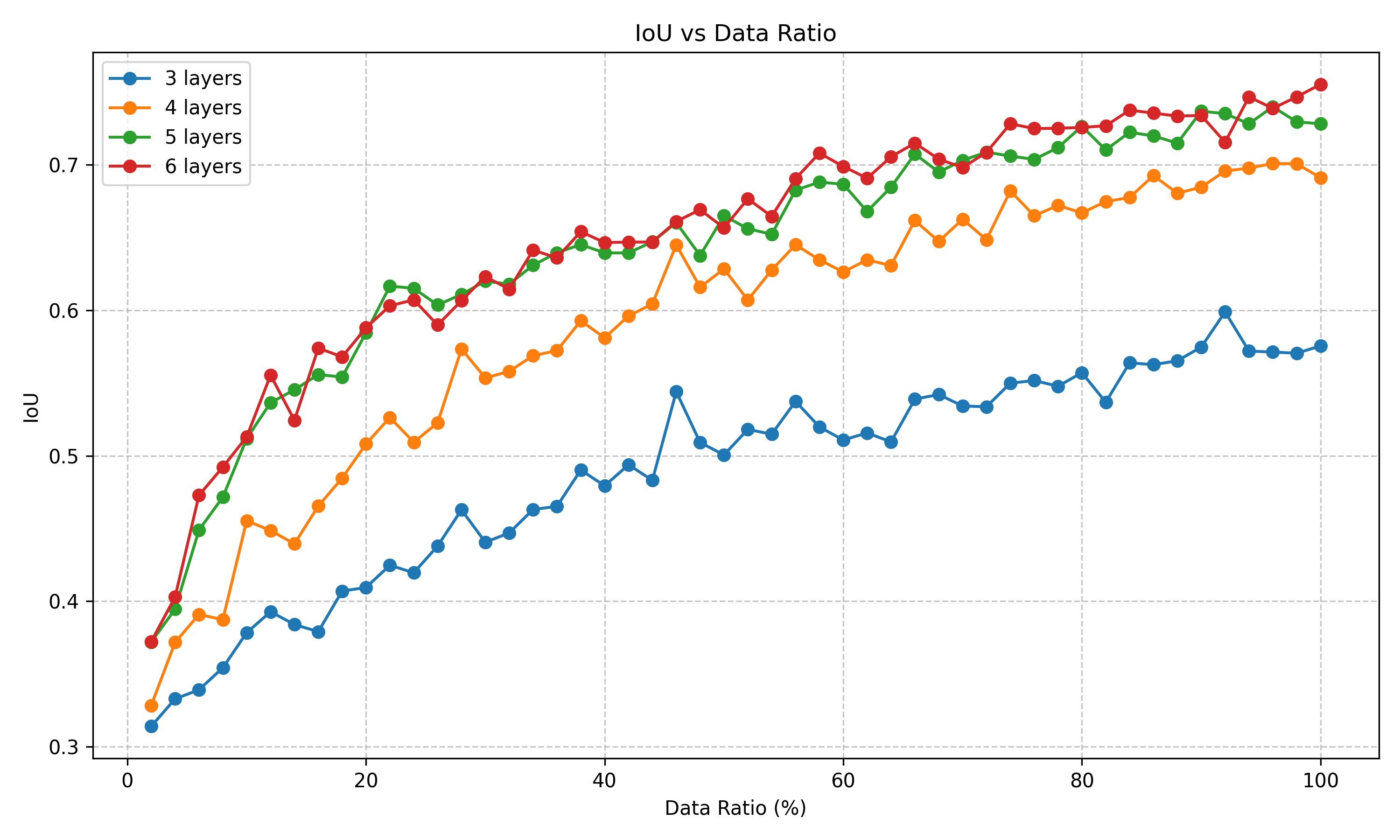

Supplement: S5 Fig — (PNG) [file pone.0339064.s005.png]

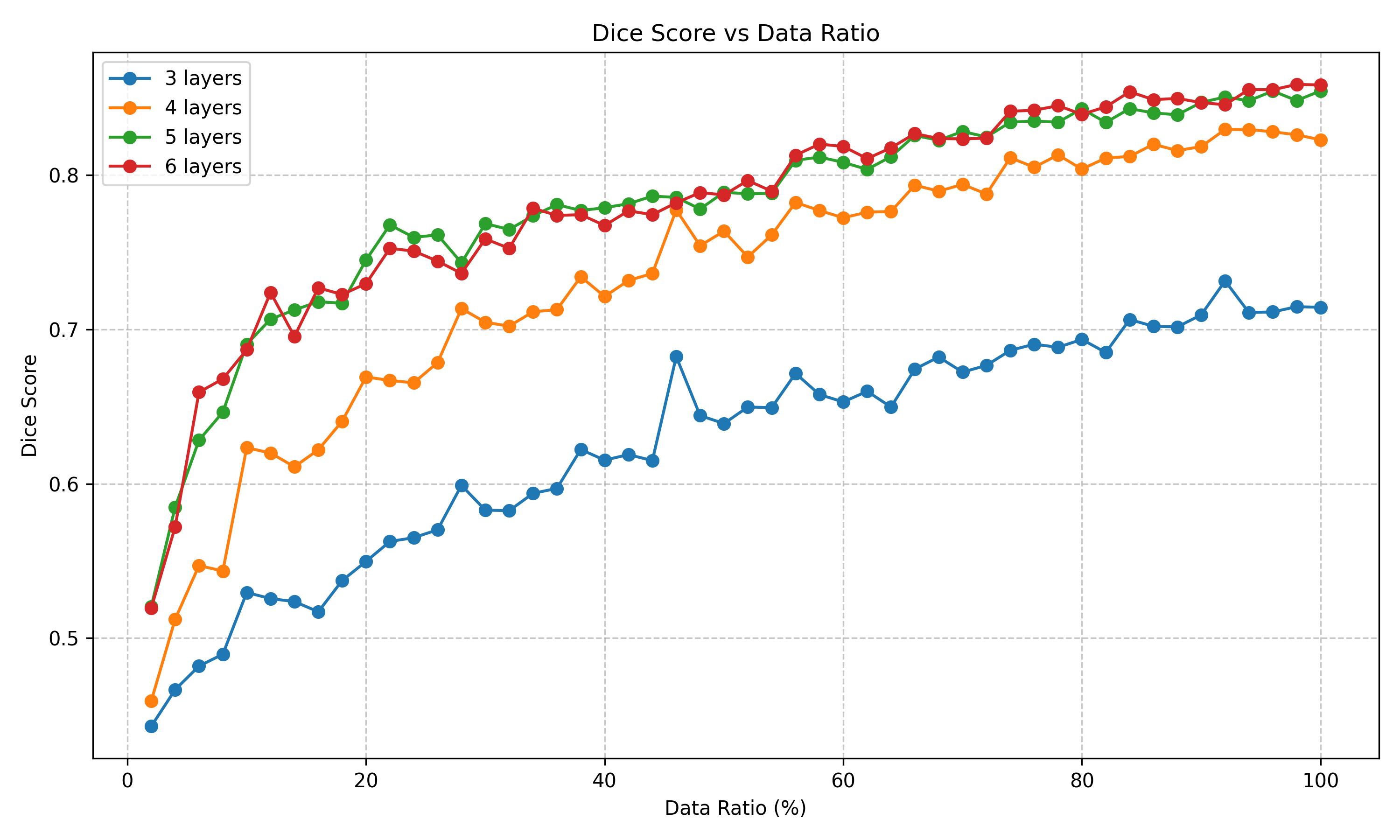

Supplement: S6 Fig — (PNG) [file pone.0339064.s006.png]

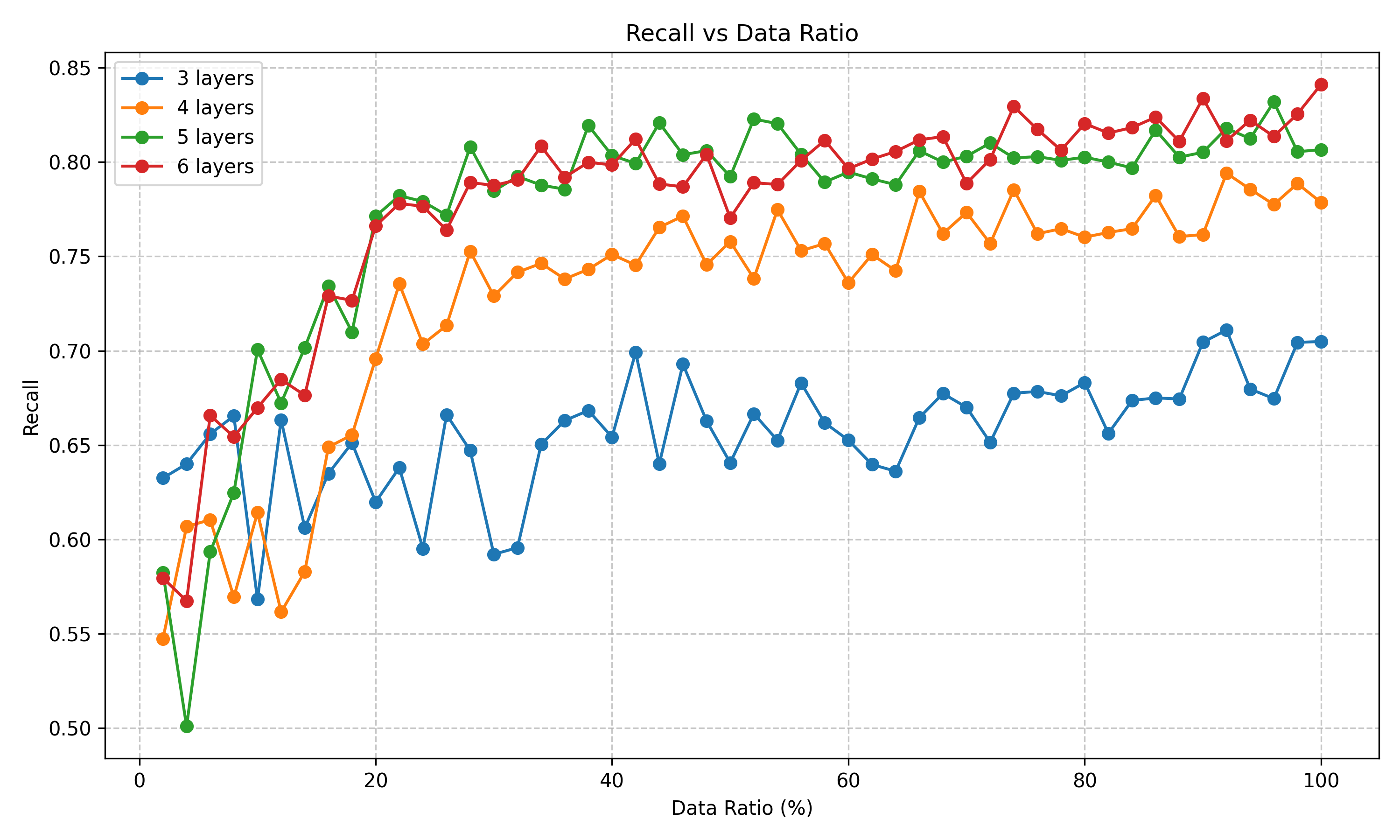

Supplement: S7 Fig — (PNG) [file pone.0339064.s007.png]

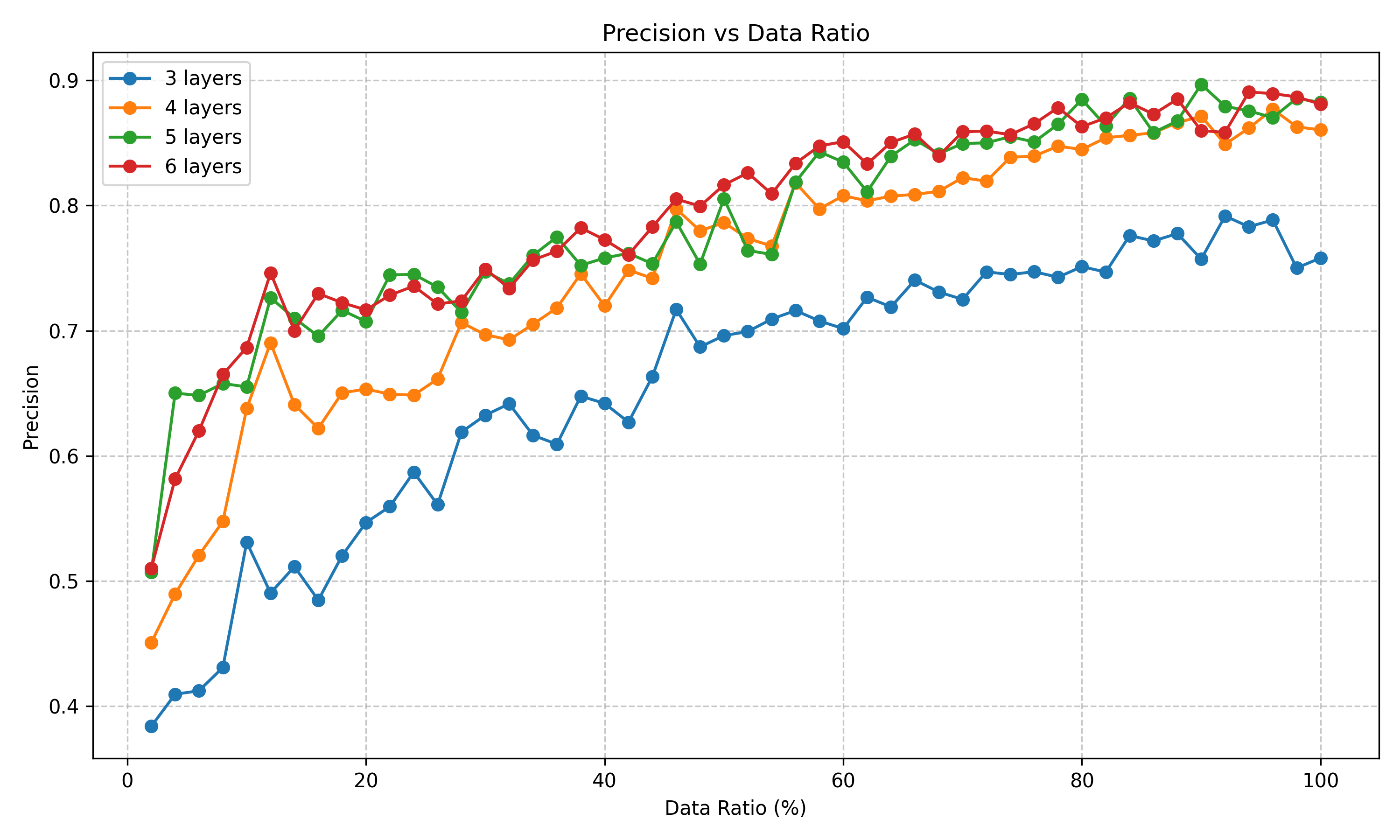

Supplement: S8 Fig — (PNG) [file pone.0339064.s008.png]

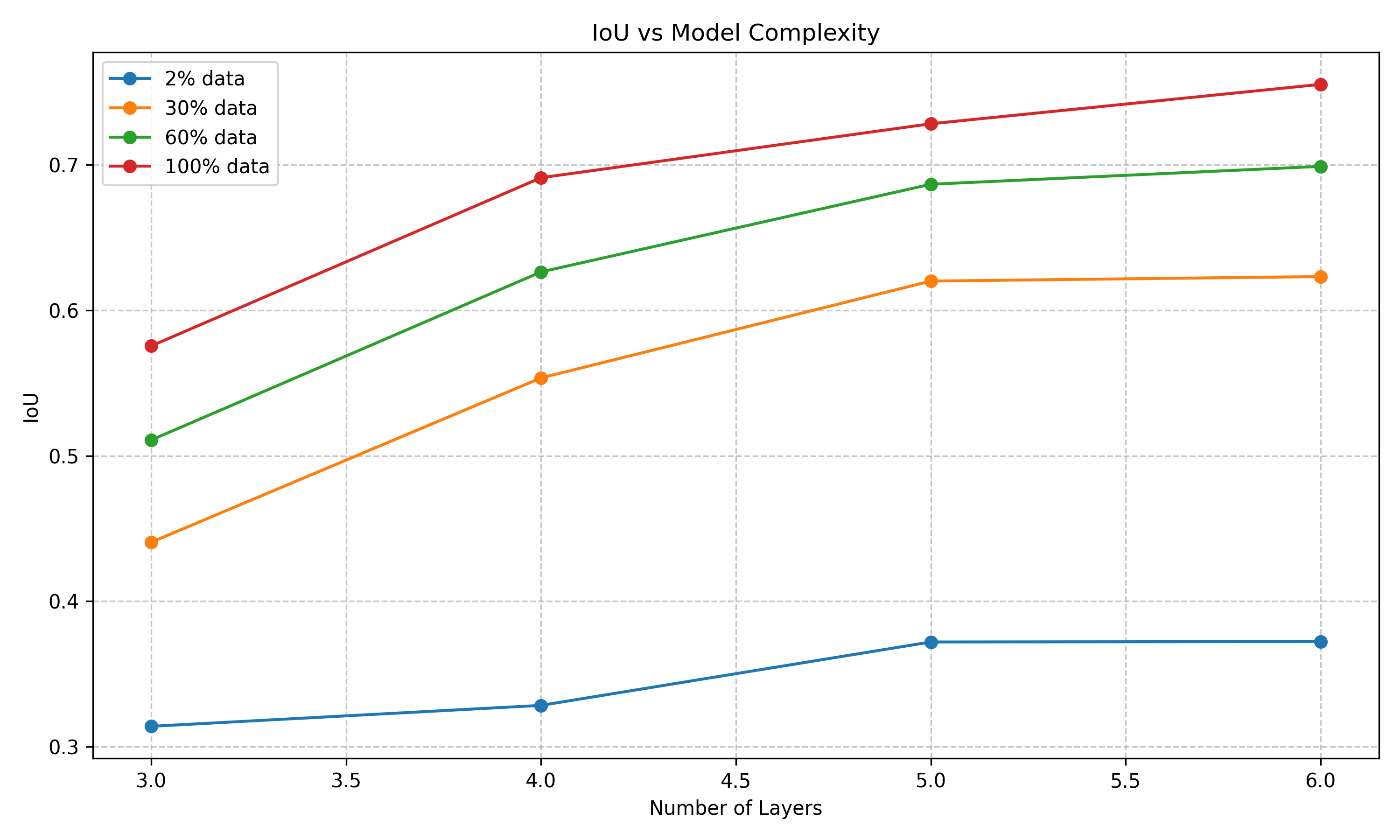

Supplement: S9 Fig — (PNG) [file pone.0339064.s009.png]

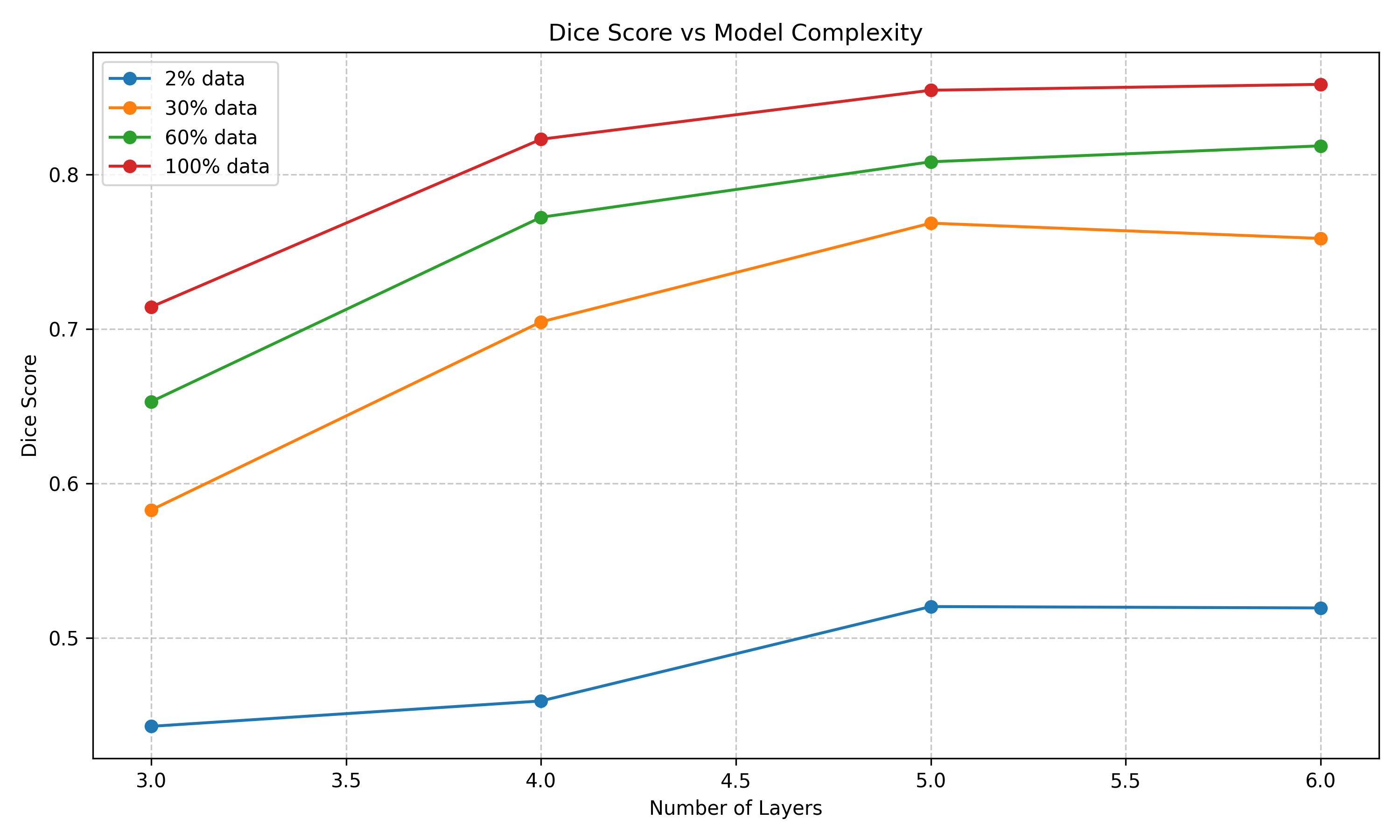

Supplement: S10 Fig — (PNG) [file pone.0339064.s010.png]

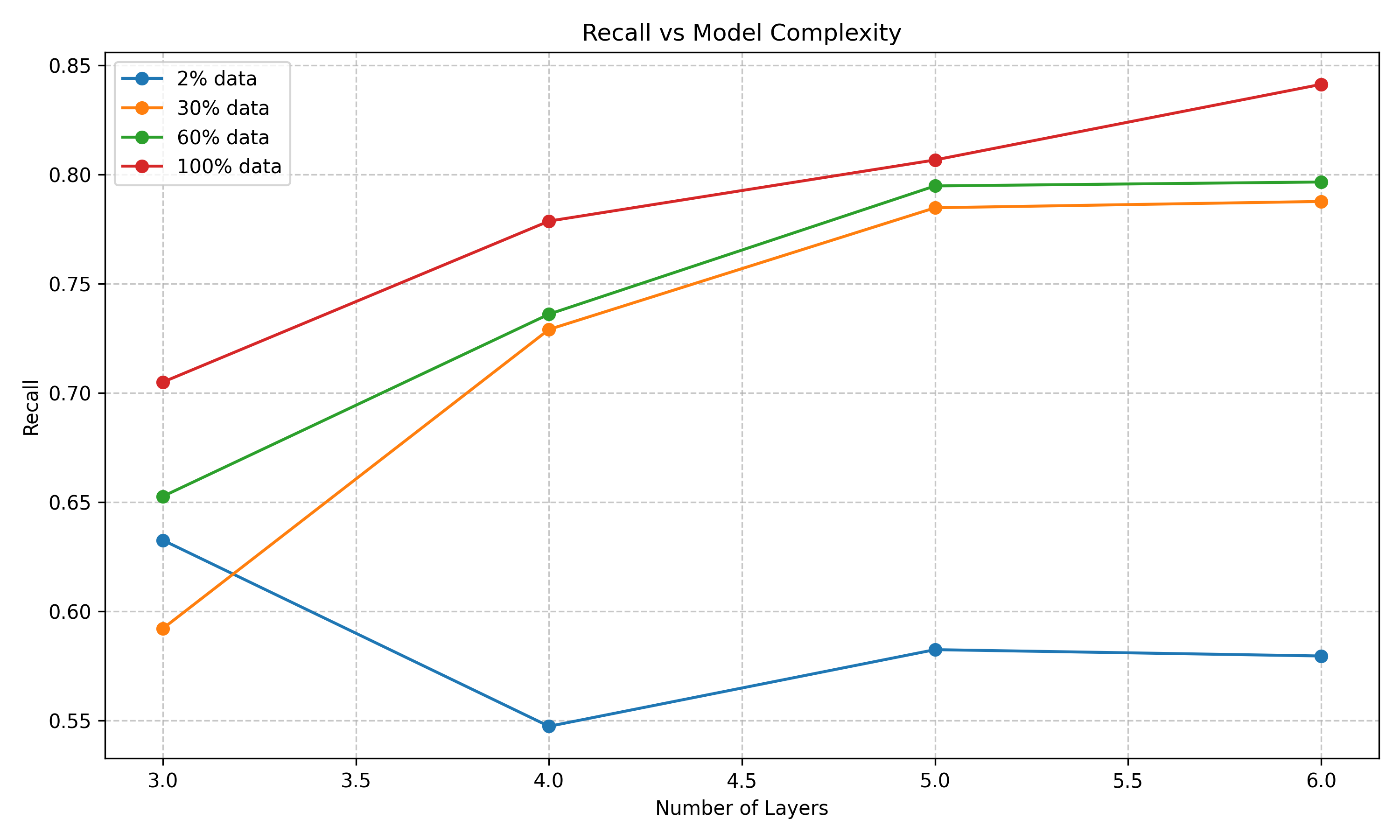

Supplement: S11 Fig — (PNG) [file pone.0339064.s011.png]

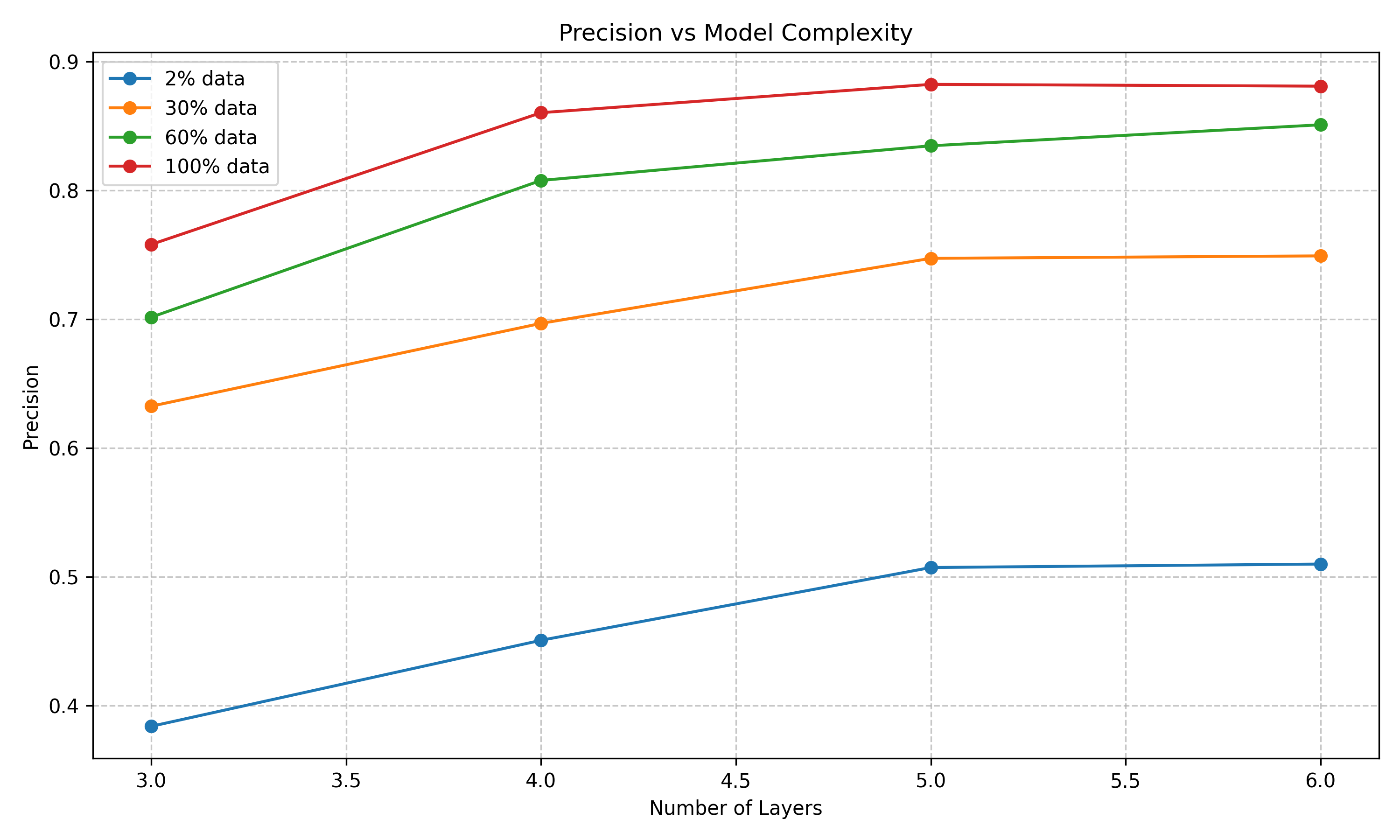

Supplement: S12 Fig — (PNG) [file pone.0339064.s012.png]

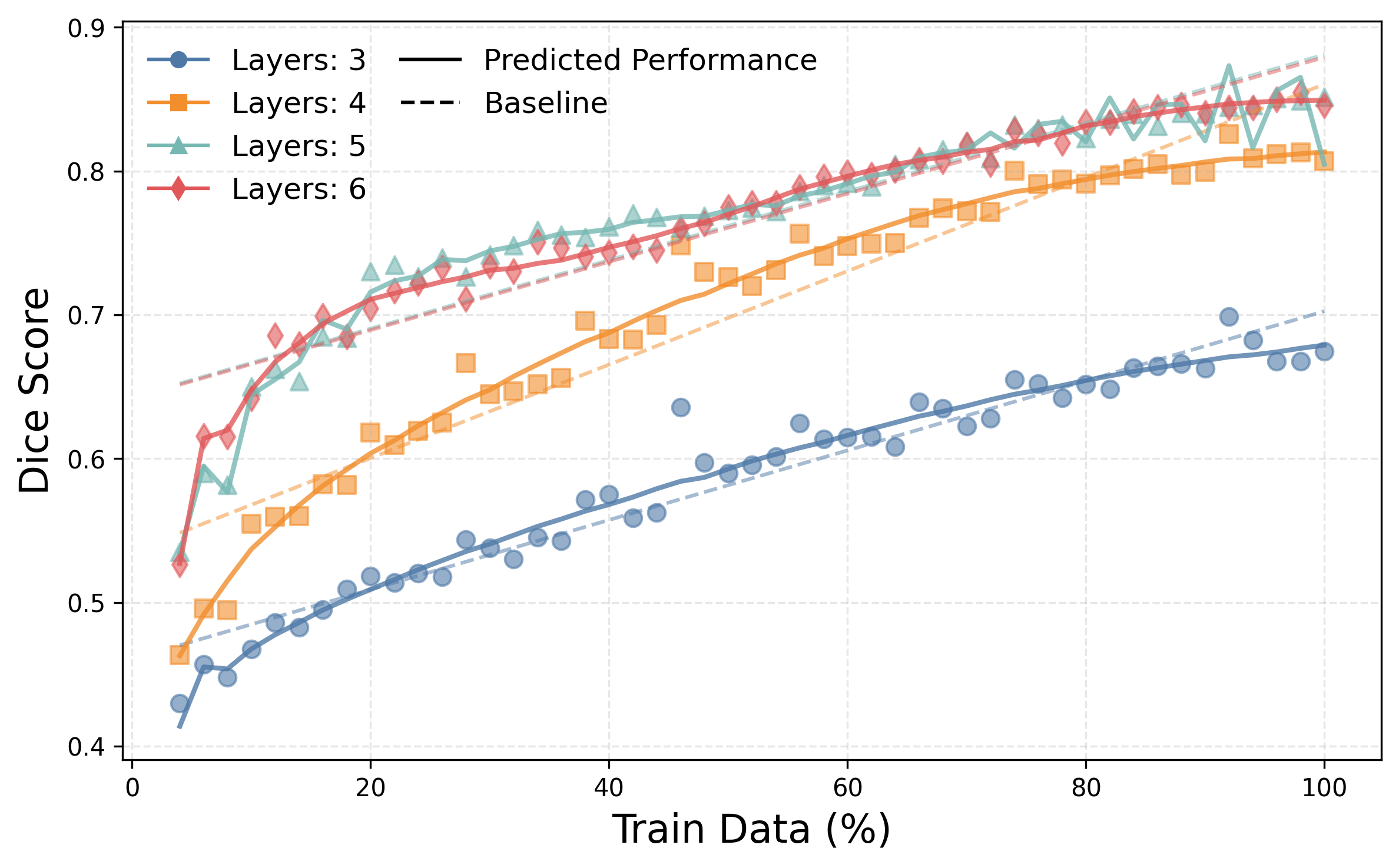

Supplement: S13 Fig — Performance prediction results generated by the uni-directional one-step LSTM model for the Kvasir-SEG dataset. The plot compares the actual DSC scores (scatter points) from the segmentation task with the model’s predicted performance (solid lines) and a linear regression baseline (dashed lines). This one-step model predicts each data point based on the sequence of preceding actual values. (TIF) [file pone.0339064.s013.tif]

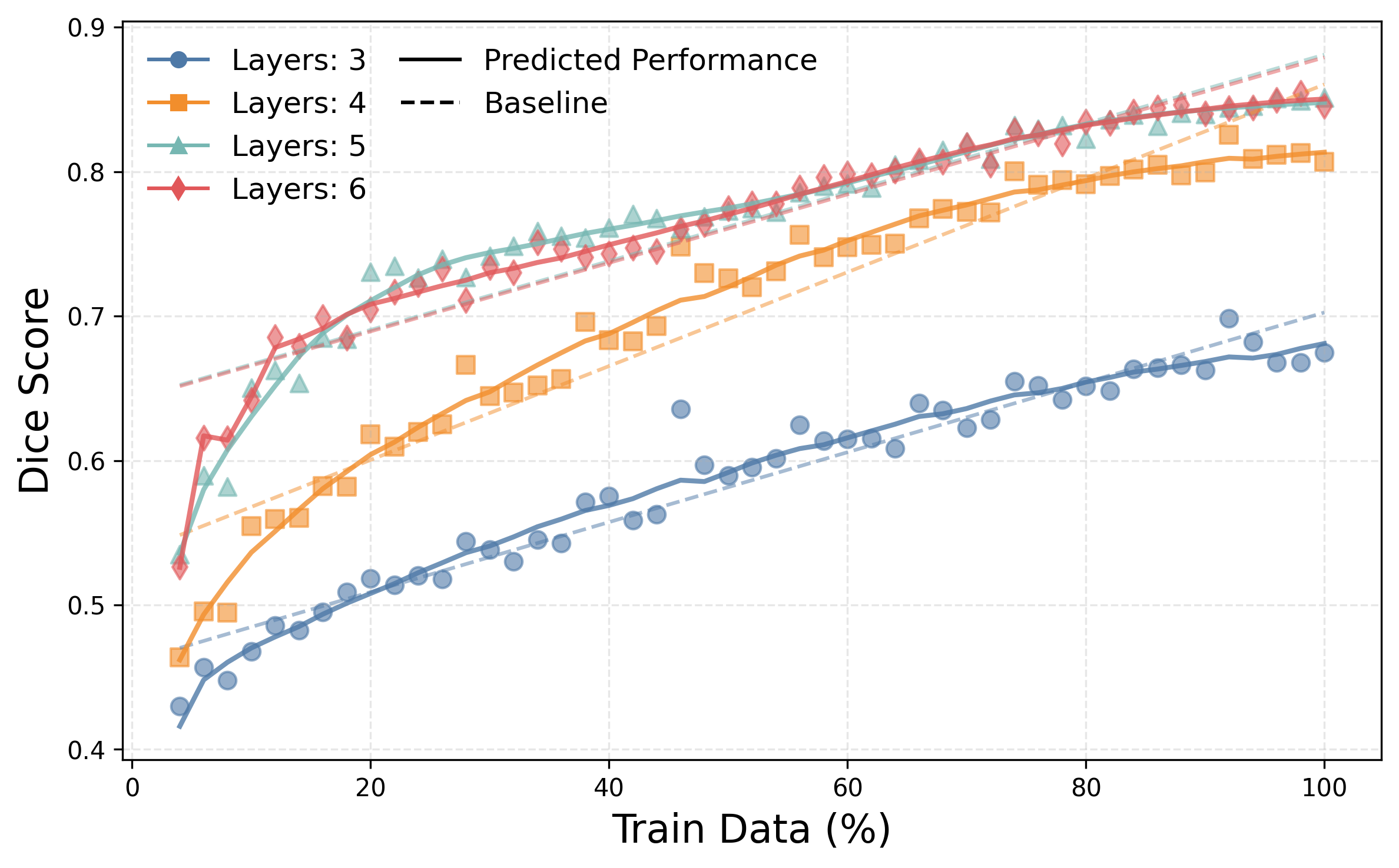

Supplement: S14 Fig — Performance prediction results from the bi-directional one-step LSTM model for the Kvasir-SEG dataset. The plot elements are consistent with previous figures: actual DSC scores (scatter points), predicted performance (solid lines), and a linear regression baseline (dashed lines). This model leverages both past and future data context to predict each point independently. (TIF) [file pone.0339064.s014.tif]

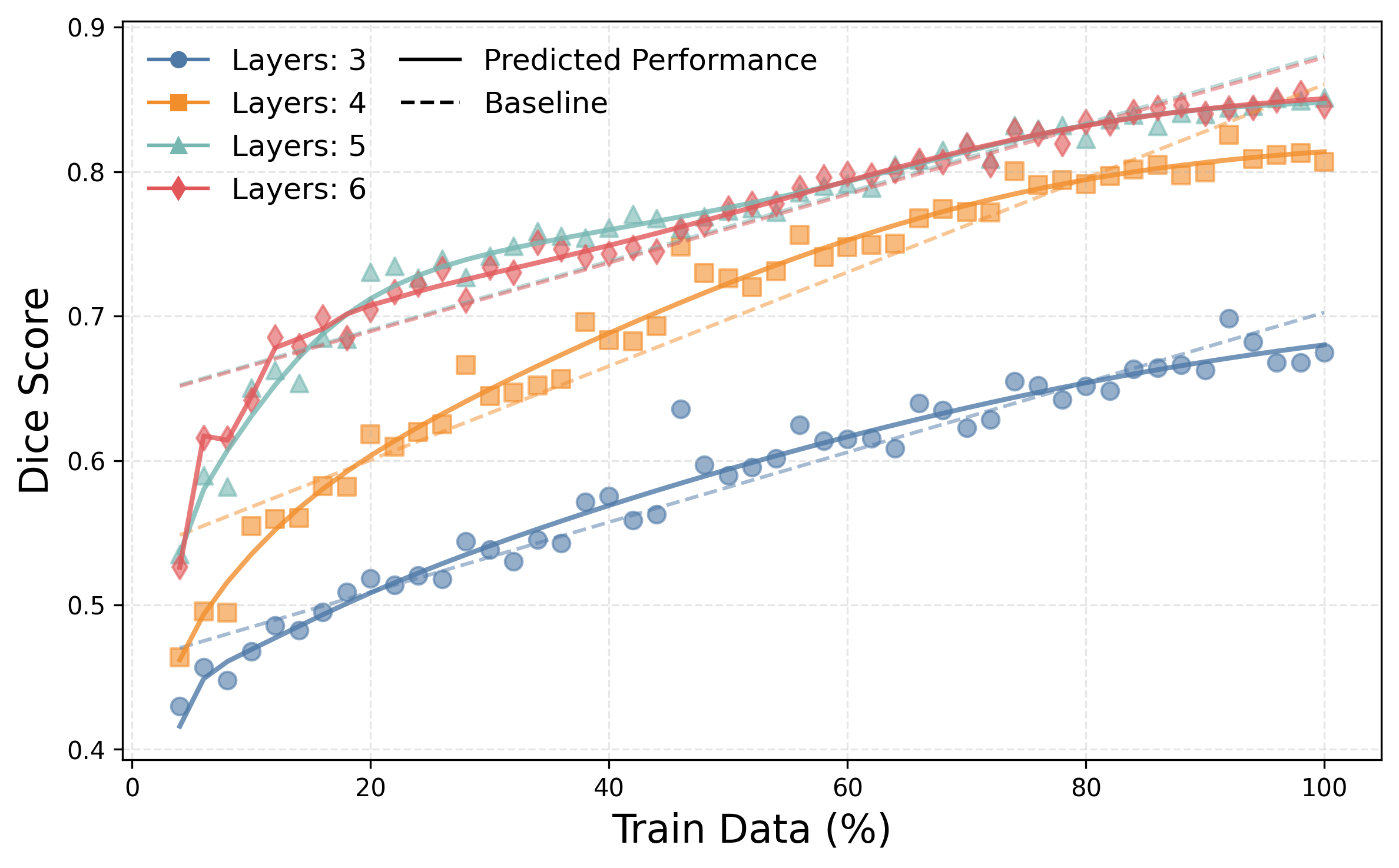

Supplement: S15 Fig — Performance prediction results from the bi-directional full-step LSTM model for the Kvasir-SEG dataset. The graph displays the actual DSC scores (scatter points) against the autoregressive predicted performance curve (solid lines) and a linear regression baseline (dashed lines). This model uses bi-directional context and its own previous predictions to forecast the entire performance trajectory. (TIF) [file pone.0339064.s015.tif]

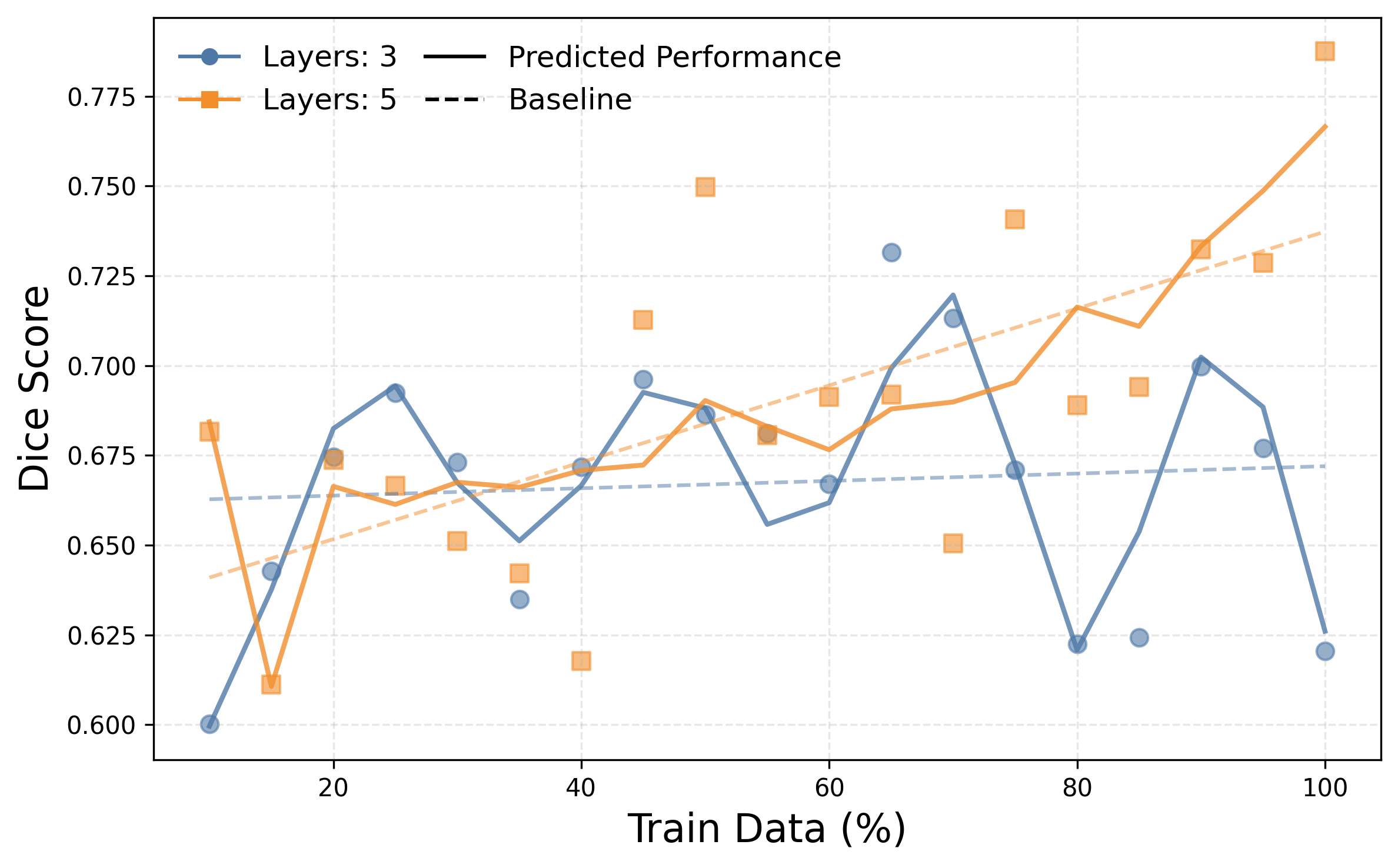

Supplement: S16 Fig — Performance prediction results generated by the uni-directional one-step LSTM model for the BraTS 2020 dataset. The plot compares the actual DSC scores (scatter points) from the segmentation task with the model’s predicted performance (solid lines) and a linear regression baseline (dashed lines). This one-step model predicts each data point based on the sequence of preceding actual values. (TIF) [file pone.0339064.s016.tif]

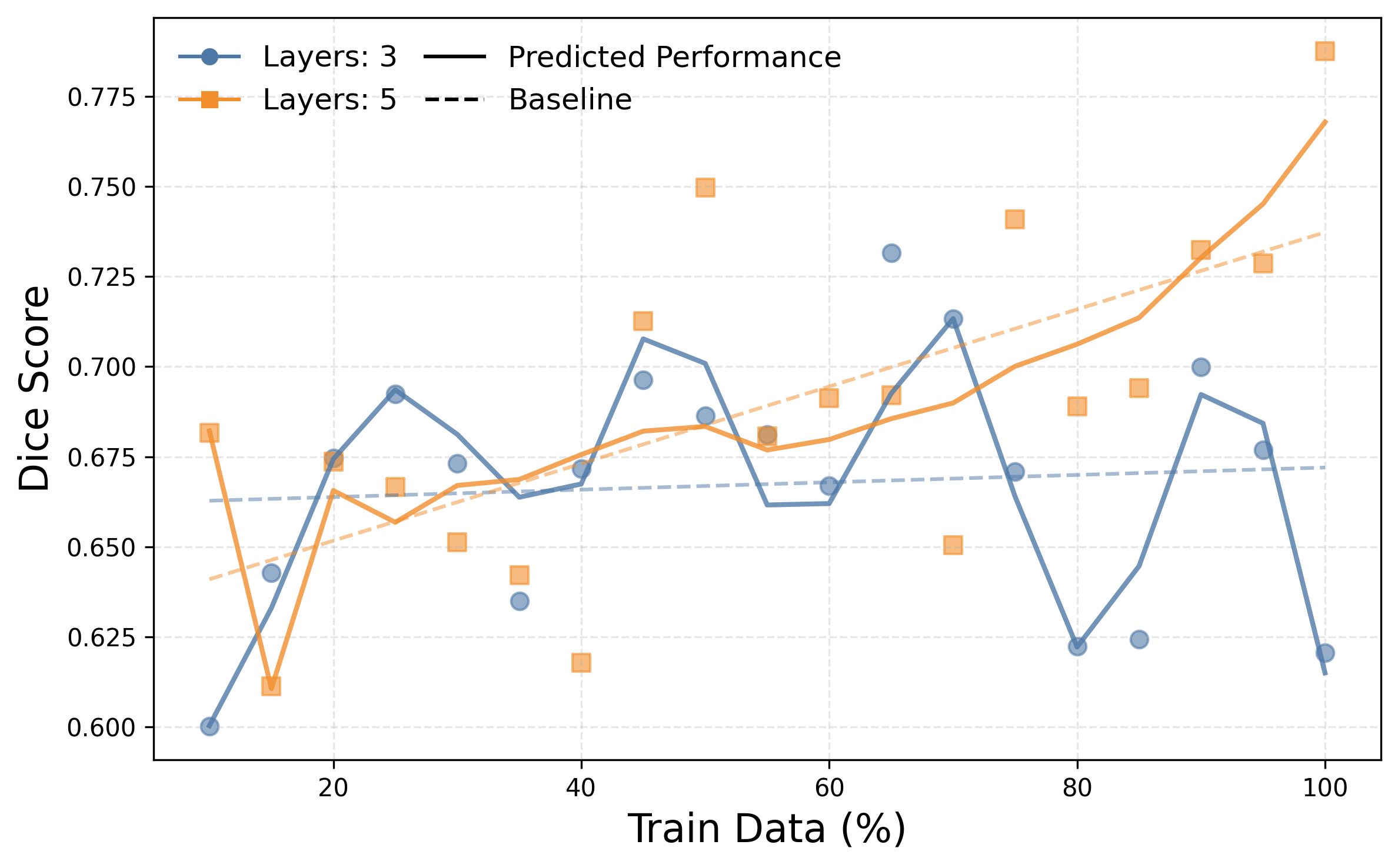

Supplement: S17 Fig — Performance prediction results from the bi-directional one-step LSTM model for the BraTS 2020 dataset. The plot elements are consistent with previous figures: actual DSC scores (scatter points), predicted performance (solid lines), and a linear regression baseline (dashed lines). This model leverages both past and future data context to predict each point independently. (TIF) [file pone.0339064.s017.tif]

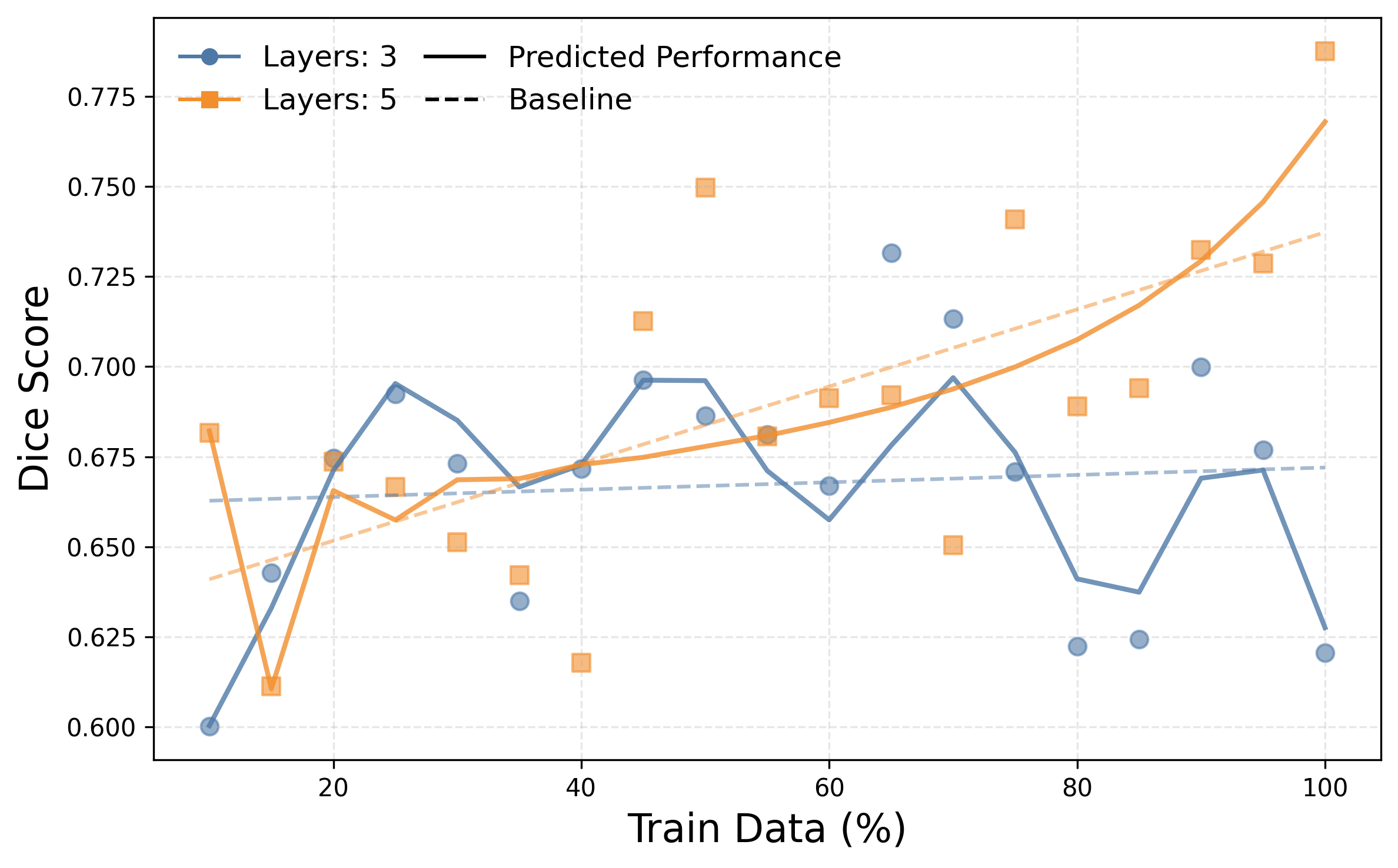

Supplement: S18 Fig — Performance prediction results from the bi-directional full-step LSTM model for the BraTS 2020 dataset. The graph displays the actual DSC scores (scatter points) against the autoregressive predicted performance curve (solid lines) and a linear regression baseline (dashed lines). This model uses bi-directional context and its own previous predictions to forecast the entire performance trajectory. (TIF) [file pone.0339064.s018.tif]
